# Towards a pragmatist dealing with algorithmic bias in medical machine learning

**DOI:** 10.1007/s11019-021-10008-5

**Published:** 2021-03-13

**Authors:** Georg Starke, Eva De Clercq, Bernice S. Elger

**Affiliations:** 1 Institute for Biomedical Ethics, University of Basel, Basel, Switzerland; 2 Center for Legal Medicine, University of Geneva, Geneva, Switzerland

**Keywords:** Artificial intelligence, Machine learning, Pragmatism, Philosophy of Science, Algorithmic bias, Fairness

## Abstract

Machine Learning (ML) is on the rise in medicine, promising improved diagnostic, therapeutic and prognostic clinical tools. While these technological innovations are bound to transform health care, they also bring new ethical concerns to the forefront. One particularly elusive challenge regards discriminatory algorithmic judgements based on biases inherent in the training data. A common line of reasoning distinguishes between justified differential treatments that mirror true disparities between socially salient groups, and unjustified biases which do not, leading to misdiagnosis and erroneous treatment. In the curation of training data this strategy runs into severe problems though, since distinguishing between the two can be next to impossible. We thus plead for a pragmatist dealing with algorithmic bias in healthcare environments. By recurring to a recent reformulation of William James’s pragmatist understanding of truth, we recommend that, instead of aiming at a supposedly objective truth, outcome-based therapeutic usefulness should serve as the guiding principle for assessing ML applications in medicine.

## Introduction

“Ethics and epistemology are always very closely related, and if we want to understand our ethics, we must look at our epistemology”, the British philosopher and novelist Irish Murdoch noted in her early essay *Metaphysics and Ethics* (Murdoch 1957, p. 113). Her statement rings eminently true with regard to ethical challenges posed by the integration of Artificial Intelligence (AI) into health care. Medical decisions are increasingly aided by recommender systems based on machine learning (ML) that support health care providers, e.g. in choosing an appropriate diagnosis or treatment for their patients. Particularly promising are programs using Deep Learning (DL) based on Artificial Neural Networks (ANN) (Topol 2019b; Esteva et al. 2019). While much research has been devoted to ML-based diagnostic classifiers, ranging from oncology to psychiatry, recent advances also promise more robust predictive measures of immediate clinical utility. For example, it has been shown that ML-based systems can identify patients suffering from chronic lymphocytic leukaemia (CLL) for whom additional immunosuppression would constitute a major risk for infection (Agius et al. 2020). Another very recent application of ML promises early predictions of circulatory failure for patients in intensive care settings (Hyland et al. 2020)—without doubt of high interest during the Covid-19 pandemic, and the list of such applications is ever increasing. For these reasons, many expect DL to revolutionize medicine and to constitute a major paradigm-shift in the practice of medicine towards an era of “Deep Medicine” (Topol 2019a).

By enhancing treatment and freeing time for patient-physician interactions, these new developments have great potential to improve clinical care. Still, they also pose numerous ethical challenges that are narrowly tied to epistemological questions concerning these programs. Of key concern are the replication and reinforcement of existing discriminatory practices by training ML programs on biased data. As is well documented, bias in medicine is pervasive, whether it is based on unconscious prejudices or rooted in systematically skewed data collection, e.g. through clinical trials carried out predominantly with male participants. In some instances, such biases can be easily detected and countered by appropriate data curation, for instance by assuring an appropriate balancing of male and female training cases. In other instances, such biases remain hidden and may prove impossible to trace, particularly if the target variable of interest, such as a diagnostic category, is based on medical convention.

Following the lead of others, we therefore turn our attention to questions of epistemology (Grote and Berens 2020), and propose a different approach which takes inspiration from philosophy of science. Following a recent reformulation of William James’s pragmatist theory of truth (Chang 2017), we argue that for some medical contexts, the debate about bias can be improved by shifting the focus of attention beyond the mere correspondence of input and target variables. Instead of clinging to a supposedly objective truth of the training data, the outcome-based clinical utility of any medical ML program should be put to the forefront. The paper proceeds in three steps: first, we introduce the notion of algorithmic bias and provide some salient examples of bias in medicine. We then provide a critique of an understanding of ground truth based on the correspondence theory of truth and suggest an alternative pragmatist reading. Lastly, we show how such an alternative view could be applied to reshape the debate about biases of medical ML. Modifying Box’s well-known maxim that all models are wrong, but some are useful (Box 1976, p. 792), we propose what one may call James’s maxim: that some models are true precisely because they are useful (James 1907 [1922], p. 204).

## Bias in medical machine learning

Bias has been at the forefront of ethical debates both in ML and in medicine for decades. The word originates from the Old Provençal word *biais*, where it described the behaviour of balls with a greater weight on one-side (OED 2020). In consequence, these balls tended to roll systematically in an oblique line into one particular direction and thus shifted the odds of a game. In the modern metaphoric sense, bias similarly describes such one-sided tendencies, usually with regard to decisions that systematically and erroneously favour or disadvantage particular decisions over others. In the context of ML, such biases take many different forms and can stem from various causes but are commonly summarized by the term *algorithmic bias.* Danks and London have suggested a useful taxonomy distinguishing between five different kinds of algorithmic bias, based on where in the design or use of a program the bias occurs (Danks and London 2017). In the context of healthcare, Thomas Ploug and Søren Holm have recently distinguished between at least three different ways in which bias could lead to discrimination in ML-based diagnostics and treatment planning (Ploug and Holm 2020). While our discussion here follows examples of algorithmic biases linked to training data, we believe that an outcome-oriented approach could equally address other forms of biases such as algorithmic processing bias, e.g. introduced through the choice of regularization or smoothing parameters (Danks and London 2017, p. 4693).

In medicine, practical examples of biases are frequently based on gender or race. They shape a plethora of vital diagnostic and therapeutic decisions, leading for example to the classic case of missed myocardial infarctions in women, which do not show the supposedly typical symptoms of a heart attack prevalent in men (Hobson and Bakker 2019). Another well-researched case concerns psychiatric decision making in black populations: black US-Americans are much more likely to be diagnosed with schizophrenia when presenting with affective symptoms than their Caucasian peers (Strakowski et al. 2003). White patients presenting with similar symptoms are in turn more likely to be diagnosed (arguably correctly) with mood disorders such as major depression. Partially, this persistent phenomenon of misdiagnosis is thought to arise from socially entrenched biases passed on by clinicians (Gara et al. 2019). Other examples include widespread misperceptions about pain management in black patients based on erroneous assumptions about physiological differences between black and white patients (Hoffman et al. 2016).

The rise of ML in medicine runs risk to exacerbate such biases, since structural racism is known to shape the collection and integration of data as well as the delivery of targeted therapeutic interventions (Geneviève et al. 2020). If, for example, one were to use the historical health records of black patients treated for schizophrenia in the US to train a diagnostic ML program, it would arguably use race as a predictor for its calculations and continue the overdiagnosis of schizophrenia in its recommendations. However, one would not only risk purporting false clinical judgements from the past in the diagnostic ML program. More problematically, if such procedures would be dignified by the common belief in the objectivity of algorithms (Galison 2019), discriminatory practices will become even more entrenched in medical practice and more difficult to address. Existing biases could become deeply hidden in the hyperparameters of an ANN, beyond the grasp of human understanding and intervention. Such algorithmic bias would skew the recommendations systematically for one particular group resulting in unfair treatment.

What about instances where we actually *do* want to discern between different socially salient groups though? A different example, where ethnicity also plays a crucial role, may serve as a useful example here. Systemic lupus erythematosus (SLE), a severe autoimmune rheumatological disease which typically affects the skin, but also many other tissues and internal organs, is known to affect more women than men and have a significantly higher prevalence in people of African, Asian or Hispanic descent (Lewis and Jawad 2017). Similar to schizophrenia, the exact underlying aetiology is, as of now, still unclear, rendering diagnosis rather difficult. With gender and ethnicity being crucial predictors for the occurrence of SLE, it would seem justified to include information on the ethnicity or gender of patients in the training data for a diagnostic program for this disease. In contrast to schizophrenia, such inclusion could be seen as warranted since it accurately mirrors true disparities between socially salient groups.^1^

Unfortunately, in most medical examples the relation between the predictor and the target variable, which shapes the so-called ground truth for an ML algorithm, is difficult to determine since the features of interest are based on medical convention. In such instances, the feature may prove to be somewhat of a shifting target, e.g. due to changing diagnostic classifications over time. Diagnostic categories in psychiatry, which have shifted drastically over the past decades, as seen easily by the consecutive revisions of the Diagnostic and Statistical Manual of Mental Disorders (DSM) over the past decades, may serve as a particularly salient example. Here, distinguishing between wrong biases that lead to misdiagnosis and erroneous treatment and justified differential treatment that mirrors true differences seems highly challenging—particularly for the many conditions and treatment options where underlying causal relations remain unclear (London 2019). Yet, simply leaving out potentially discriminatory labels such as gender or ethnicity as input variables can apparently not solve the problem either. After all, to our best knowledge, ethnicity and gender seem to play a role for diagnostic, therapeutic and prognostic purposes in many diseases, as the example of SLE highlights. So, what may we do about these unclear instances of bias, in lieu of a clear standard against which to measure it?

## Bias and the pragmatist theory of truth

A common strategy to address the problem of bias is to further the transparency of ML models (Mittelstadt et al. 2019; Vayena et al. 2018). The underlying assumption is that greater transparency will render algorithmic bias easier to detect and help understand a program’s erroneous decisions, so that one can correct the algorithm’s mistakes and avoid bias by curating the input variables accordingly. For many instances this solution can be sufficient, e.g. to identify so-called Clever Hans predictors that base a ML program’s classification strategy on irrelevant correlations. A good example for such a misleading predictor is a program basing the classification of an image as ‘horse’ on a source tag in the training images for horses (Lapuschkin et al. 2019). Based on such ill-curated input data, the program will erroneously assume that all future testing images displaying this source tag depict horses, largely independent from the image’s actual content. Increasing a program’s transparency, one could identify the source tag as a decisive, yet meaningless factor for the decision-making process, enabling an ex-post correction. Transferred to the clinical example, if an explainable program allows seeing that a diagnosis of schizophrenia is at least partly based on a person’s skin colour, anyone commanding trained judgement could notice this as erroneous and account for it.

In medicine, checking a program’s decisions is not just a technical challenge though. Returning to the two clinical examples, both schizophrenia as well as SLE constitute heuristic constructs based on a number of diagnostic criteria, while the underlying aetiology remains subject to scientific debate. Put differently, there is no valid gold standard for establishing a ground truth—a wide-spread problem in medicine, that concerns all medical fields, even those with supposedly clear-cut pathological correlates such as oncology (Adamson and Welch 2019). After all, most biological differences only become meaningful in medicine if they are correlated with symptoms and complaints—a process that is by definition highly conventional and ultimately also pragmatic. How may one distinguish in these instances between irrelevant correlations, shaped by human prejudice and convention, and causally relevant, yet currently unknown, predictors, e.g. based on genetic factors that are more prevalent in certain groups? One seemingly easy remedy to avoid discriminatory practices would be to forego the potentially problematic category altogether. For example, one could simply leave out ethnicity or gender as an input to achieve a non-discriminatory program.

Prima facie, this would safeguard the World Medical Association’s Declaration of Geneva, prohibiting considerations of ethnic origin, gender or race to interfere with medical duties (Parsa-Parsi 2017). However, this approach runs into two major problems. First, it has been shown to be very challenging to implement, since the category which should not influence the training data may be inferred by other seemingly innocent input data, with ZIP-codes and socio-economic status being amongst the most obvious (Mehrabi et al. 2019). Second, for specific instances, e.g. unequal distribution of genetic disease predisposing factors in humans from different races, some form of positive discrimination may seem warranted, justifying the inclusion of ethnicity in the training data. Again, the example of SLE can serve as a useful example here. As discussed above, SLE mainly affects populations which constitute minorities in most Western countries. If ethnicity was categorically excluded as a potential input in the training data, it would be more difficult to obtain a correct diagnosis for this vulnerable population. Rendering the diagnosis of a disease such as SLE less accurate in minority populations by disregarding race could easily be regarded as discriminatory.

One solution to this conundrum may lie in taking a step back and looking at the relation between input and output space anew, which as the terminology of the field already indicates, is supposed to be a truth relation. The way such truth is usually constructed assumes the classic understanding of truth, namely the correspondence theory of truth.^2^ Commonly ascribed to Aristotle, this theory posits that a proposition “p” is true if and only if it corresponds to some fact. Put differently, according to this theory truth describes a relation between a truth bearer such as a proposition or a judgement and a truth-maker, such as an observable fact in the empirical world. In the context of ML, the ground truth relation can be similarly described as a mapping of different spaces onto each other.

In its simplest form, such mapping occurs between an observable input space and an intended output or decision space. In addition to these, some authors have proposed adding a so-called construct space in-between these two, capturing unobservable, yet meaningful predictors, as a third mediating space to formally address structural bias (Friedler et al. 2016). Seemingly, one could also apply their framework to the medical cases at hand here: the input space would contain clinical observations, e.g. symptoms or clinical findings, whereas the decision space would contain the recommended treatment. The construct space could be found in the agreed upon diagnostic criteria that are presumed to be of relevance by the medical community. Unfortunately, for the many and highly relevant cases in medicine where such causal relations between the different spaces continue to be unknown, such mapping remains highly spurious. As long as we do not know, for example, the causal link between brain-based pathology causing psychotic episodes, the presumed diagnostic construct of schizophrenia and the therapeutic mechanism of specific antipsychotic drugs, any such mapping will remain to some extent arbitrary and open to challenge.

However, there are also other ways to construe truth, that look at practices rather than at propositions and which may be better suited to the medical contexts at hand. One suggestive model is the pragmatic theory of truth, the best-known version of which was formulated by William James in 1907 (James 1907 [1922]). James, who tellingly had received medical training himself, famously stressed the practical value of statements. Turning against both rationalist and empiricist conceptions, James argued for defining their truth in terms of utility. In his lectures *Pragmatism: A New Name for Some Old Ways of Thinking*, James famously espoused this “instrumental view of truth”, describing it as “any idea upon which we can ride, so to speak; any idea that will carry us prosperously from any one part of our experience to any other part, linking things satisfactorily, working securely, simplifying, saving labour” (James 1907 [1922], p. 58). His challenge to the correspondence theory finally culminates in his frequently cited statement that “you can say of it then either that “it is useful because it is true” or that “it is true because it is useful”. Both these phrases mean exactly the same thing” (James 1907 [1922], p. 204).

Ever since their publication, these claims have subjected James to myriads of strong criticism, due to his supposedly antirealist stance (Capps 2019). Notwithstanding this critique, we take it that a pragmatic approach may be worth reconsidering for construing truth in medical ML and, in particular, to address some of the ethical challenges posed by algorithmic bias. However, to do so, it may be more convenient to turn to a contemporary reading of James from the philosophy of science, which already addresses the criticism of James’s account. The philosopher of science Hasok Chang, whose work has already been successfully employed to address other challenges in the context of nosology (Kendler 2012), prominently advocates a Jamesian pragmatist model of epistemology in the sciences. Chang reframes James’s model to provide an understanding of truth based on operational coherence, rooted in action. While Chang explicitly rejects a correspondence theory of truth, his notion of coherence also “goes beyond consistency between propositions; rather, it consists in various actions coming together in an effective way towards the achievement of one’s aims” (Chang 2017, p. 109).

Applied to the medical context, such aims can entail simpler tasks such as immobilizing a broken bone with a plaster cast to promote its healing process, or highly complex aims requiring many different actors. The recent development of workable tracing apps to contain the spread of Covid-19 may serve as an example here. Within such given contexts, true statements are those necessary to achieve one’s aims. As Chang puts it: “A statement is true in a given circumstance if (belief in) it is needed in a coherent activity” (Chang 2017, p. 113)*.* Based on the coherent system in question, different and possibly contradictory statements may have been adopted as true in the history of science insofar as they produced or improved certain kinds of knowledge for particular aims.

Given this historical contingency of science, one may be tempted to disregard the notion of truth in science altogether. While James’s original approach may seem to support such a relativist stance, rendering the world dependent upon the interests of its describer (Putnam 1994, p. 448), Chang’s model of operational coherence does not severe the crucial connection between knowledge and reality in a similar fashion, precisely because it demands to be rooted in empirical facts: “operational coherence cannot be achieved in an arbitrary fashion by decree, wishful thinking, or mere mutual agreement. On the contrary, in order to do things successfully in the world, we need to have an understanding and mastery of our surroundings. It is operational coherence, not the mirage of correspondence, through which the mind-independent world is actually brought to bear on our knowledge” (Chang 2017, p. 112).

Leaving more fundamental philosophical questions aside, this implies two crucial practical benefits for its application to medical ML. First, it does not undermine the powerful notion of scientific truth in the public sphere—a notion that seems to be intricately related to public trust in science (Shapin 1995). Second, it supports retaining the vocabulary of (ground) truth as a technical term for the necessary pairing of input and output variables (Gil-Fournier and Parikka 2020), without making overly ambitious claims about medical truths—which as we have seen are frequently subject to contingent conventions. As Chang notes, his approach of “[c]hecking for pragmatic necessity may not live up to some overblown image of a philosophical test, but it is how we get on in science, and in the rest of life too” (Chang 2017, p. 115). In the following, we will show what this may mean practically in the context of medical ML.

## Bias in medical ML: a pragmatist approach

We argue that a pragmatic understanding of “ground truth” can be highly informative for algorithmic bias in medical ML. Clearly, the overarching aim of the medical community needs to concur with the Ancient Hippocratic idea: the aim of medicine is to work for the benefit of the sick, to cure them or at least make them better. In our opinion, these general ambitions provide a rather clear purpose for our collective epistemic practices—even though the exact determination of its content will be subject to much debate for different applications in different diseases and diverse clinical contexts. To enable an open debate about the clinical utility of particular programs and their potential risks, it is worth trying to consider medical ML in terms of operational coherence guided by specific medical aims. Outcome-based therapeutic usefulness should serve as the guiding principle for their design, not a recourse to a supposedly objective truth based on a static correspondence theory.^3^

Returning to the two clinical examples of schizophrenia and SLE, we can now apply this model to the context of bias in medical ML. In the case of schizophrenia, it seems clear that the diagnostic practice of US psychiatrists of readily diagnosing their black patients with schizophrenia did not further their well-being but may in fact have resulted in maltreatment and harmful medication and should hence be abolished. In comparison, the case of lupus provides quite a different picture. Here, a differentiation based on ethnicity could contribute to patients’ well-being, if it increases diagnostic accuracy resulting in adequate treatment; it would thus be (to some degree) warranted to be included in the construction of ground truth.

There are at least three points of major concern that could be levelled against this position. First, one could argue that a utility-based account of ground truth is not adequate for all medical applications. And indeed, for some instances, mere data curation may be sufficient. When causal links between clinical observation, diagnostic construct and available treatment are clearly established, interpretable or explainable ML models can help to identify misleading or unnecessary input data. As a classical example one could think of diabetes mellitus type 1 (DM1), where the destruction of pancreatic beta-cells provides a clear aetiology that can be linked to clinical observations such as recurrent hyperglycaemia, the diagnostic construct of DM1 with certain predicted measurements under fasting, and the suggested treatment with insulin (Stegenga 2018, p. 26). However, as we have shown, this is far from the rule in medicine—not only in specialties with a notoriously challenging nosology such as psychiatry but also in e.g. internal medicine or dermatology. As Alex London has argued, the unknown aetiology of many medical conditions thus demands a primate of accurate diagnosis and treatment over explainability in the context of medical ML (London 2019).^4^

A second and potentially more serious problem concerns the measurement and operationalisation of clinical utility.^5^ After all, a pragmatist evaluation of medical ML based on clinical utility will need to be based on clear and operationalizable criteria to avoid an infinite circle and yield a useful guide for developers and regulating bodies. At the same time, one should also aim to avoid an overly prescriptive and potentially paternalistic definition of clinical utility, without patient involvement. The increasing use of short- and long-term Patient-Reported Outcome Measures (PROMs) aims to address this conundrum (McClimans 2010), but relies on inherently subjective criteria (Alexandrova 2017, pp. 135–138). Of course, this is a problem that not only applies to medical ML, but evidence-based medicine more generally, and defies a simple and general answer. In consequence, heading Jacob Stegenga’s advice that “(t)he instruments employed in clinical research should measure patient-relevant and disease-specific parameters” (Stegenga 2015, p. 62), it may be fruitful to return to the concrete examples of SLE and schizophrenia.

In the case of schizophrenia, the very construct of the disorder, as laid out in ICD-10 and DSM5, largely relies on clinical observations by the attending psychiatrist, which are based on verbal self-reports from the patient. Outcome measures of schizophrenia are thus always multi-faceted attempts to grasp this complex reality—including neurobiological measures, drop-out from antipsychotic treatment, hospitalisations, structured symptom scales and patient-reported outcomes such as personal well-being—and have changed drastically since the disorder was first described by Kraepelin in 1896 (Burns 2007). In addition, since the course of the disorder seems to be influenced heavily by social context (Leff et al. 1992), outcome measures need to be adapted to specific contexts. In comparison, SLE seems to pose fewer problems, with standardized and congruent diseases activity scores, based on clinical observations such as seizures and objectifiable measures like proteinuria (American College of Rheumatology 2004). Similarly, standardized PROMs for SLE have successfully been adapted for different cultural contexts (Navarra et al. 2013; Kaya et al. 2014; Bourré-Tessier et al. 2013), so the utility of an intervention for SLE could tentatively be evaluated based on a combination of these instruments. Still, as these examples highlight, choosing an appropriate outcome-measure will be context- and disease-specific, and always open for debate—in the context of ML as much as for other medicinal products. It is thus crucial that studies are explicit about their operationalization and measurement of clinical benefit, to allow patients and physicians to arrive at an informed choice regarding their individual use.

A third challenge relates directly to ethics. If we are to follow clinical utility as the single most important criterion for the evaluation of medical applications of ML, this could be misinterpreted as a call for a simplistic reading: that maximizing the benefit for the majority of patients justifies disregarding the needs of a potentially vulnerable minority. Our approach is different insofar as we deem it necessary to embrace explicit criteria for algorithmic fairness, derived from moral philosophy. We consider John Rawls’ difference principle to be a potential contender, which prioritizes the well-being of those who are worst off (Rawls 1999, pp. 132–134).

Such a principle may be enacted by constrained optimization algorithms that maximize clinical utility but also need to satisfy other set conditions, based on evaluations calculated separately for various subgroups (Corbett-Davies et al. 2017). There is extensive research from the area of fair ML that demonstrates how Rawls’ theory of justice can be practically incorporated (Lundgard 2020), e.g. as a constraint for classification (Jabbari et al. 2017; Joseph et al. 2016) or as loss minimization (Hashimoto et al. 2018). Of course, implementing a fairness constraint for ML algorithms requires intricate ethical judgements, e.g. concerning who counts as worse-off than others, a point which will often be contentious. In addition, there may be good reasons to implement fairness constraints that go beyond Rawls, “artificial intelligence’s favorite philosopher” (Procaccia 2019, cit. in Lundgard 2020, p. 3). For instance, in many ML applications ensuring the benefit of the patient will require a careful evaluation of different layers of vulnerabilities, as Paolo Corsico has recently argued with view to psychosis (2020). In the medical context, such approaches could translate to regulatory rules that demand tests whether an ML program performs worse in ethnic minorities, in terms of clearly defined outcome-measures, and denies approval to those which do.

## Lessons for the evaluation of medical ML

For the design of medical ML programs, developers should thus focus on ex-post corrections of particular ML programs in medicine and evaluate a program’s performance based on the relative treatment outcome within certain vulnerable populations.^6^ The examples of schizophrenia and SLE highlight this. Clearly, the pragmatic benchmark of a ML-based diagnostic program would be the treatment success that results from applying it to patients.^7^ Let us consider two options: (1) implementing a ML program designed to be blind to ethnicity and (2) designing the ML algorithm in a way that it explicitly or implicitly incorporates ethnicity as input variable in the training data. Embracing a pragmatic approach, the decision for using program 1 or 2 would focus on the clinical results which either program brings about. If, for example, algorithm 2 results in better outcomes in both black and white populations, then the differential treatment would be useful and hence, in the pragmatic sense, true. Based on our current knowledge, one would expect to find this result for the case of SLE. However, if algorithm 1 results in better treatment outcomes in both groups, than a differential treatment is apparently harmful, biased and should be disregarded, as may be the case in schizophrenia.

Still, based on the concept of operational coherence, these a priori assumptions require empirical testing. After all, one could similarly envision a contrary case, where an algorithm explicitly taking into account ethnicity performs better in terms of fairness for diagnosing schizophrenia. For example, depending on the design, ethnicity could be used as a correcting factor that counters the known overdiagnosis of schizophrenia among black patients. Here, transparency will be key for a critical reassessment of the assumptions underlying each particular program. Still, the choice of ML program will ultimately need to be adjudicated by its tangible clinical benefit.

While this may run counter to preferences in the machine learning community to focus on ex-ante mechanisms to ensure fairness, such an approach has been proven to be highly efficient in addressing discriminatory behaviour of algorithms, based for example on gender stereotypes (Zhao et al. 2017). In medicine, some form of such ex-post tests on fairness could be integrated in clinical trials, conducted so that a specific program receives approval by regulatory bodies such as the US Food and Drug Administration (FDA) (He et al. 2019). This would also imply that both short- and long-term outcome of the ML system are tested and its safety and utility evaluated in different phases, transitioning from few healthy volunteers to large clinical trials in the target population (Paulus et al. 2016).

We thus believe that a pragmatist approach focusing on a program’s output would also constitute a viable and realistic way to address disparities for medical applications where ex-ante considerations are potentially impossible due to limited etiological knowledge and the often-conventional nature of medical practice. If we thereby move closer to accepting that also ML will replicate and not remove the shifty and often pragmatic ground of medicine, this could be a safeguard to avoid an overselling of the promises of medical ML. Such a viewpoint may further render us humbler and more willing to accept the epistemic limitations and historical contingency of much contemporary medical knowledge (Stegenga 2018, p. 185–187). Thus, instead of focusing on potentially fruitless nosological speculations, we should instead try and privilege a focus on operational coherence, centred around the most crucial criterion in the medical domain: the betterment of the patient.

## Conclusion

In this paper, we have argued for a pragmatic construction of truth in the context of supervised medical ML. Following two clinical examples with unknown etiological underpinnings, we have defended a position that stresses the importance of rigorous ex-post tests for medical ML programs to tackle harmful biases. Instead of aiming for a potentially unobtainable objective truth, developers, clinicians and regulators should pragmatically focus on clinical utility for specific socially-salient groups when evaluating the fairness of a ML system—as well as the many other ethical and value-laden considerations that Char et al. (2020) have recently identified, such as: who devises these programs, based on which assumptions, and with which aims? If a pragmatist account of bias can help to clear the view for such questions, this may be all the more reason to embrace it.

## Footnotes

1 Of course, this is not to make any metaphysical claims or advocate a naturalistic understanding of diseases. We merely want to highlight that categories such as gender or ethnicity, intricately related to social and environmental factors, can often serve as useful predictors for diagnostic or therapeutic decisions.

2 We do not intend to make any claims concerning the factual epistemological beliefs of ML developers here. Many may in fact embrace an instrumental understanding of truth, whether explicitly or implicitly. However, the term ground truth itself and its historical origins in geography and aerial reconnaissance, referring to the physical ground, seem to suggest a relation of correspondence (Gil-Fournier and Parikka 2020).

3 With regard to diagnostic hypotheses, Stanley and Nyrup have recently made a similar point drawing on Charles Sanders Peirce, and suggested to conceptualise the diagnostic process as a form of strategic reasoning (2020).

4 While we agree with London (2019), who recommends prioritizing accuracy over explainability in the context of medical ML, we believe that a pragmatic focus on clinical utility may be better suited to stress the value-ladenness of ML systems as well as their embeddedness in a pragmatic context.

5 We would like to thank two anonymous reviewers for their help in making this point more explicit.

6 It should be noted though that such outcome-based evaluation of different programs would need to refer to their entire design process, including the choice of respective training data.

7 With regard to diagnostic decisions based on ML, we take it that these will also largely affect treatment outcomes since they determine the indication of therapeutic intervention.

## References

[CR1] Adamson Adewole S, Gilbert Welch H (2019). Machine Learning and the cancer-diagnosis problem—No gold standard. New England Journal of Medicine.

[CR2] Agius Rudi, Brieghel Christian, Andersen Michael A, Pearson Alexander T, Ledergerber Bruno, Cozzi-Lepri Alessandro, Louzoun Yoram, Andersen Christen L, Bergstedt Jacob, von Stemann Jakob H (2020). Machine learning can identify newly diagnosed patients with CLL at high risk of infection. Nature Communications.

[CR3] Alexandrova Anna (2017). A philosophy for the science of well-being.

[CR46] American College of Rheumatology (2004). The American College of Rheumatology response criteria for systemic lupus erythematosus clinical trials: Measures of overall disease activity. Arthritis & Rheumatology.

[CR4] Bourré-Tessier Josiane, Clarke Ann E, Mikolaitis-Preuss Rachel A, Kosinski Mark, Bernatsky Sasha, Block Joel A, Jolly Meenakshi (2013). Cross-cultural validation of a disease-specific patient-reported outcome measure for systemic lupus erythematosus in Canada. The Journal of Rheumatology.

[CR5] Box George E. P (1976). Science and statistics. Journal of the American Statistical Association.

[CR6] Burns Tom (2007). Evolution of outcome measures in schizophrenia. Britih Journal of Psychiatry Supplement.

[CR7] Capps John, Zalta EN (2019). The pragmatic theory of truth. The Stanford encyclopedia of philosophy.

[CR8] Chang Hasok (2017). Operational coherence as the source of truth. Proceedings of the Aristotelian Society.

[CR9] Char Danton S, Abràmoff Michael D, Feudtner Chris (2020). Identifying ethical considerations for machine learning healthcare applications. American Journal of Bioethics.

[CR10] Corbett-Davies, Sam, Emma Pierson, Avi Feller, Sharad Goel, and Aziz Huq. 2017. Algorithmic decision making and the cost of fairness. In *Proceedings of the 23rd ACM SIGKDD international conference on knowledge discovery and data mining*, 797–806.

[CR11] Corsico Paolo (2020). Psychosis, vulnerability, and the moral significance of biomedical innovation in psychiatry. Why ethicists should join efforts. Medicine, Health Care and Philosophy.

[CR12] Danks, David, and Alex J. London. 2017. Algorithmic bias in autonomous systems. In *Proceedings of the 26th international joint conference on artificial intelligence*, 4691–4697.

[CR13] Esteva Andre, Robicquet Alexandre, Ramsundar Bharath, Kuleshov Volodymyr, DePristo Mark, Chou Katherine, Cui Claire, Corrado Greg, Thrun Sebastian, Dean Jeff (2019). A guide to deep learning in healthcare. Nature Medicine.

[CR14] Friedler, Sorelle A., Carlos Scheidegger, and Suresh Venkatasubramanian. 2016. On the (im)possibility of fairness. arXiv preprint https://arXiv.org/1609.07236.

[CR15] Galison Peter, Brockman John (2019). Algorists dream of objectivity. Possible minds: 25 ways of looking at AI.

[CR16] Gara Michael A, Minsky Shula, Silverstein Steven M, Miskimen Theresa, Strakowski Stephen M (2019). A naturalistic study of racial disparities in diagnoses at an outpatient behavioral health clinic. Psychiatric Services.

[CR17] Geneviève Lester D, Martani Andrea, Shaw David, Elger Bernice S, Wangmo Tenzin (2020). Structural racism in precision medicine: Leaving no one behind. BMC Medical Ethics.

[CR18] Gil-Fournier Abelardo, Parikka Jussi (2020). Ground truth to fake geographies: Machine vision and learning in visual practices. AI & Society.

[CR19] Grote Thomas, Berens Philipp (2020). On the ethics of algorithmic decision-making in healthcare. Journal of Medical Ethics.

[CR20] Hashimoto, Tatsunori B, Megha Srivastava, Hongseok Namkoong, and Percy Liang. 2018. Fairness without demographics in repeated loss minimization. arXiv preprint https://arXiv.org/1806.08010.

[CR21] He Jianxing, Baxter Sally L, Jie Xu, Jiming Xu, Zhou Xingtao, Zhang Kang (2019). The practical implementation of artificial intelligence technologies in medicine. Nature Medicine.

[CR22] Hobson Philippa, Bakker Julia (2019). How the heart attack gender gap is costing women's lives. British Journal of Cardiac Nursing.

[CR23] Hoffman Kelly M, Trawalter Sophie, Axt Jordan R, Norman Oliver M (2016). Racial bias in pain assessment and treatment recommendations, and false beliefs about biological differences between blacks and whites. Proceedings of the National Academy of Sciences of the United States of America.

[CR24] Hyland Stephanie L, Faltys Martin, Hüser Matthias, Lyu Xinrui, Gumbsch Thomas, Esteban Cristóbal, Bock Christian (2020). Early prediction of circulatory failure in the intensive care unit using machine learning. Nature Medicine.

[CR25] Jabbari, Shahin, Matthew Joseph, Michael Kearns, Jamie Morgenstern, and Aaron Roth. 2017. Fairness in reinforcement learning. In *International conference on machine learning*: PMLR.

[CR26] James, William. 1907 [1922]. *Pragmatism: A new name for some old ways of thinking*. New York: Longmans, Green & Co.

[CR27] Joseph, Matthew, Michael Kearns, Jamie Morgenstern, Seth Neel, and Aaron Roth. 2016. Rawlsian fairness for machine learning. arXiv preprint https://arXiv.org/1610.09559.

[CR28] Kaya Arif, Goker Berna, Cura Elife Senem, Tezcan Mehmet Engin, Tufan Abdurrahman, Mercan Rıdvan, Bitik Berivan, Haznedaroglu Seminur, Ozturk Mehmet Akif, Mikolaitis-Preuss Rachel A (2014). Turkish lupusPRO: Cross-cultural validation study for lupus. Clinical rheumatology.

[CR29] Kendler Kenneth S, Kendler Kenneth S (2012). Epistemic iteration as a historical model for psychiatric nosology: Promises and limitations. Philosophical issues in psychiatry II: Nosology.

[CR30] Lapuschkin Sebastian, Wäldchen Stephan, Binder Alexander, Montavon Grégoire, Samek Wojciech, Müller Klaus-Robert (2019). Unmasking Clever Hans predictors and assessing what machines really learn. Nature Communications.

[CR31] Leff J, Sartorius N, Jablensky A, Korten A, Ernberg G (1992). The International pilot study of schizophrenia: Five-year follow-up findings. Psychological Medicine.

[CR32] Lewis Myles J, Jawad Ali S (2017). The effect of ethnicity and genetic ancestry on the epidemiology, clinical features and outcome of systemic lupus erythematosus. Rheumatology.

[CR33] London Alex J (2019). Artificial intelligence and black-box medical decisions: Accuracy versus explainability. Hastings Center Report.

[CR34] Lundgard, Alan. 2020. Measuring justice in machine learning. arXiv preprint https://arXiv.org/2009.10050.

[CR35] McClimans Leah (2010). A theoretical framework for patient-reported outcome measures. Theoretical Medicine and Bioethics.

[CR36] Mehrabi, Ninareh, Fred Morstatter, Nripsuta Saxena, Kristina Lerman, and Aram Galstyan. 2019. A survey on bias and fairness in machine learning. arXiv preprint https://arXiv.org/1908.09635.

[CR37] Mittelstadt, Brent, Chris Russell, and Sandra Wachter. 2019. Explaining explanations in AI. In *FAT* ‘19: Proceedings of the conference on fairness, accountability, and transparency*, 279–288.

[CR38] Murdoch Iris, Pears David (1957). Metaphysics and ethics. The nature of metaphysics.

[CR39] Navarra SV, Tanangunan RMDV, Mikolaitis-Preuss RA, Kosinski M, Block JA, Jolly M (2013). Cross-cultural validation of a disease-specific patient-reported outcome measure for lupus in Philippines. Lupus.

[CR40] OED (2020). Oxford english dictionary online.

[CR41] Parsa-Parsi Ramin Walter (2017). The revised declaration of Geneva: A modern-day physician's pledge. JAMA.

[CR42] Paulus Martin P, Huys Quentin J, Maia Tiago V (2016). A roadmap for the development of applied computational psychiatry. Biological Psychiatry: Cognitive Neuroscience and Neuroimaging.

[CR43] Ploug Thomas, Holm Søren (2020). The right to refuse diagnostics and treatment planning by artificial intelligence. Medicine, Health Care and Philosophy.

[CR44] Putnam Hillary (1994). Sense, nonsense, and the senses: An inquiry into the powers of the human mind. Journal of Philosophy.

[CR45] Rawls John (1999). A theory of justice.

[CR47] Shapin Steven, Bulger R, Meyer Bobby E, Fineberg HV (1995). Trust, honesty, and the authority of science. Society’s choices: Social and ethical decision making in biomedicine.

[CR48] Stanley Donald E, Nyrup Rune (2020). Strategies in abduction: Generating and selecting diagnostic hypotheses. Journal of Medicine and Philosophy.

[CR49] Stegenga Jacob (2015). Measuring effectiveness. Studies in History and Philosophy of Biological and Biomedical Sciences.

[CR50] Stegenga Jacob (2018). Medical nihilism.

[CR51] Strakowski Stephen M, Keck Paul E, Arnold Lesley M, Collins Jacqueline, Wilson Rodgers M, Fleck David E, Corey Kimberly B, Amicone Jennifer, Adebimpe Victor R (2003). Ethnicity and diagnosis in patients with affective disorders. Journal of Clinical Psychiatry.

[CR52] Topol Eric J (2019). Deep medicine: How artificial intelligence can make healthcare human again.

[CR53] Topol Eric J (2019). High-performance medicine: The convergence of human and artificial intelligence. Nature Medicine.

[CR54] Vayena Effy, Blasimme Alessandro, Glenn Cohen I (2018). Machine learning in medicine: Addressing ethical challenges. PLoS Medicine.

[CR55] Zhao, Jieyu, Tianlu Wang, Mark Yatskar, Vicente Ordonez, and Kai-Wei Chang. 2017. Men also like shopping: Reducing gender bias amplification using corpus-level constraints. arXiv preprint https://arXiv.org/1707.09457.

